# Olive Fertility as Affected by Cross-Pollination and Boron

**DOI:** 10.1100/2012/375631

**Published:** 2012-07-31

**Authors:** A. Spinardi, D. Bassi

**Affiliations:** Department of Plant Production, Università degli Studi di Milano, 20133 Milano, Italy

## Abstract

Self-compatibility of local olive (*Olea europaea* L.) accessions and of the cultivars “Frantoio” and “Leccino” was investigated in Garda Lake area, northern Italy. Intercompatibility was determined for “Casaliva,” “Frantoio,” and “Leccino,” as well as the effects of foliar Boron applications (0, 262, 525, or 1050 mg*·*L^−1^) applied about one week before anthesis on fruit set, shotberry set, and on *in vitro* pollen germination. Following self-pollination, fruit set was significantly lower and the occurrence of shot berries significantly higher than those obtained by open pollination. No significant effect of controlled cross-pollination over self-pollination on fruit set and shotberry set was detectable. B treatments increased significantly fruit set in “Frantoio” and “Casaliva” but not in “Leccino.” B sprays had no effect on shotberry set, suggesting that these parthenocarpic fruits did not strongly compete for resources allocation and did not take advantage of increased B tissue levels. Foliar B application enhanced *in vitro* pollen germination, and the optimal level was higher for pollen germination than for fruit set. Our results highlight the importance of olive cross pollination for obtaining satisfactory fruit set and the beneficial effect of B treatments immediately prior to anthesis, possibly by affecting positively the fertilisation process and subsequent plant source-sink relations linked to fruitlet retention.

## 1. Introduction

Olive (*Olea europea* L.) trees usually bloom profusely and produce pollen in great abundance. Nevertheless, only 10% to 15% of the about 500.000 flowers produced by a mature olive tree will set fruit [1, 2]. The major reduction in the flower and fruit population occurs from 5 to 7 weeks after full bloom [3]. Shedding of staminate flowers begins soon after full bloom [4] and partially overlaps the abscission of unfertilised perfect flowers triggered by pollination and/or fertilisation of adjacent flowers, taking place in the days after petal drop [5]. Most fertilised ovaries abscission, occurring after two weeks following full bloom, is affected by substrate competition among growing fruits and between fruits and other sinks [6] that proceeds until about six weeks after anthesis. After petals fall, about 25% of the ovaries are retained, but only a small percentage of fruits reaches maturity. Griggs et al. [7] have defined a good commercial yield as even only about 1% of the total number of flowers setting fruit and remaining until harvest. Thus, massive drop of flowers and fruits just after bloom is a major responsible of the low efficiency in final setting fruit and product yield.

Olive tree is a wind-pollinated allogamous species and self-incompatibility in olive has been reported by many authors (among others: [8, 9]). Self-incompatibility reaction causes an inhibition or a delay in pollen tube growth resulting in a lower percentage of fertilisation. Olive cultivars show different levels of self-incompatibility, ranging between self-compatible and totally self-infertile behaviours. Conflicting reports exist about the classification of pollen compatibility in some cultivars, and contradictory results were obtained in different locations and years [10]. The need for cross-pollination in self-incompatible cultivars and its beneficial effect on cultivars considered self-compatible [11] can also vary among years [7]. Climatic and environmental conditions immediately preceding and during bloom and fruit set can significantly affect the degree of self-compatibility and yield. High temperatures (30°–35°C) have inhibitory effects on pollen germination and pollen tube growth [12, 13] and increase the level of self-incompatibility and incompatibility in some cross-pollination combinations [14]. Moreover, pollen tube growth under self-fertilisation is generally slower than under cross-fertilisation. Thus, adverse weather conditions and orchard management practices, like lack of water, reduce the effective pollination period (EPP), that is, the window of time when pollination may result in fertilisation [15, 16] and can reduce the ability of flowers to be self-fertilised. Quero et al. [17] assume that slower tube elongation under self-pollination makes ovule longevity critical, as the embryo sac begins to degenerate before the pollen tube reaches it. Optimal orchard management practices should, therefore, be intended to extend EPP and maximise fertilisation success by increasing pollen tube growth rate.

Boron (B) effect on *in vitro* normal pollen germination and tube elongation has been well documented [18, 19]. B accumulates in flower buds and flower parts [20]. B levels are higher in floral than in vegetative tissues, suggesting a specific involvement of B in the reproductive process.

Furthermore, abscission of olive flowers after fertilisation can be mainly attributed to competition for nutrient supply among developing fruits and between fruits and actively growing shoots acting as sinks [3, 4, 6]. In some fruit species, flowers drop has been linked to B deficiency [21]. In some cases, foliar B application resulted in significant fruit set and crop yield increases [22–24].

The purpose of this paper was to determine self- and cross-compatibility of some olive cultivars in the area of Garda Lake, northern Italy, as well as to study the effects of foliar B applications on fruit set and on *in vitro* pollen germination. 

## 2. Materials and Methods

### 2.1. Plant Material

The research was conducted on a olive germplasm collection located at Puegnago, on the southwest shore of Garda Lake in northern Italy. This region is one of the northernmost olive growing districts in the world. Irrigation, fertilisation, and management practices of the varietal plot were comparable to those of a well-managed, commercial orchard.

The local olive accessions “Casaliva,” “Gargnà,” “Mitria,” and “Regina” and the more broadly cultivated “Frantoio” and “Leccino” were selected for investigations on self-fertility. Intercompatibility was determined for “Casaliva,” “Frantoio,” and “Leccino,” together with the influence of treatments on *in vitro* pollen germination.

### 2.2. Climatic Conditions

Before and during bloom, climatic data were recorded near the collection field (Latitude: 45°32′N, Longitude: 10°31′E, Altitude: 142 m) in 2008 and 2009; furthermore, data collected from 2000 to 2009 were also taken in account.

### 2.3. Controlled Pollination and Fruit Set Determination

Self-compatibility evaluation was performed in 2008. Ten healthy, densely blooming branches, uniform in length and distribution throughout the canopy were selected on four replicate trees, and the total number of flowers was determined. A few days before anthesis, the tagged shoots were isolated by paper bags, which were removed only after petal fall. On the same four trees, ten more branches were tagged as open pollination controls.

Cross-pollination was performed in 2008 on “Casaliva,” “Frantoio,” and “Leccino.” For this purpose, four trees were selected for each cultivar. Thirty uniform branches were tagged and isolated by bags: ten for pollen collection and twenty (ten per pollen) for cross-pollination trials. Flowers were not emasculated, in order to reproduce field conditions. Open flowers were hand pollinated twice by a fine camelhair brush at 50% and about 90% of open flowers, respectively. The pollen was applied quickly removing the bags under optimal weather conditions, that is, no wind and rain. 

Fruit set was checked 90 days after full bloom. Incidence of parthenocarpic and commercially useless fruit, called shotberries, was also determined.

To evaluate the degree of self-fertility in the cultivars studied, the index of self-incompatibility (ISI), an indicator introduced by Lloyd [25] and then used in tree breeding researches by Zapata and Arroyo [26], was calculated as the ratio between fruit set in self- and cross- or open pollination and classified as >1 = self-compatible; >0.2 < 1 = partially self-incompatible; <0.2 = mostly self-incompatible; 0 = completely self-incompatible.

### 2.4. Boron Foliar Applications

In 2009, B as Solubor DF (Na_2_B_8_O_13_ *·*4H_2_O; BASF, trademark Borax Europe Limited) containing 17.5% B was applied, as a foliar spray, about one week before anthesis at four concentrations (0, 262, 525, or 1050 mg·L^−1^), according to other treatments in the literature [20, 21], in 800 L*·*ha^−1^ of water, by a handgun operated sprayer, to trees exhibiting no vegetative symptoms of B deficiency. The mean B concentration in the leaves of these trees was 20–30 mg kg^−1^ dry matter. Each treatment was tested on ten uniform branches on four replicate trees of “Casaliva,” “Frantoio,” and “Leccino.” Each branch was sprayed to drip. A few days after the spray the treated shoots were bagged. The open pollination was replaced by a “controlled” open pollination treatment using a 5 : 3 : 2 mixture of “Casaliva,” “Frantoio,” and “Leccino” pollen, as this ratio approaches the actual acreage of these most widespread cultivars. Controlled pollination was carried out at three different times, with 50–60%, 70–80%, and 90–100% of open flowers.

### 2.5. *In Vitro* Pollen Germination

Pollen harvested was hydrated at 100% relative humidity for 12 h and then incubated in Petri dishes on a medium containing 15% sucrose and 0.8% agar [27]. Pollen germination was recorded after 6 h at 25°C as the percentage of germinated pollen in a total of 1000 grains from different areas of the plate. Each pollen sample was replicated three times. Pollen was considered to have germinated if pollen tube length was at least twice as long as the diameter of the grain [27]. Samples were observed by Optical Microscope (Leica DMR) and acquired by Leica DC300F Digital Camera. 

### 2.6. Statistical Analysis

Data were analysed by SPSS software (SPSS Inc., Chicago, IL.). Analysis of variance was performed for each variable. The minimum differences for significance were obtained using the Duncan's range values for the maximum number of means to be compared.

## 3. Results

### 3.1. Comparison of Climatic Parameters

**Figure 1:**
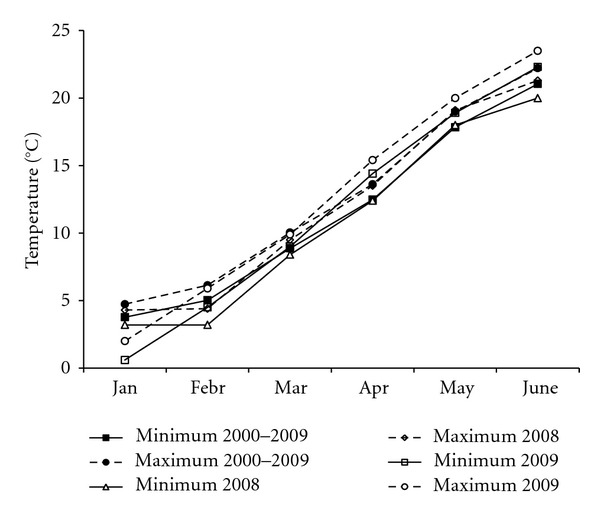
Total monthly means of daily maximum and minimum temperatures in Puegnago, on the southwest side of Lake Garda, northern Italy, from January to June in 2008 and 2009 compared to the long-term averages.

Figure 1 shows the monthly averages of daily minimum and maximum temperatures during winter 2008 and spring 2009 in Puegnago together with the medium term averages. Although the flowering period lasted from end of May to mid-June, winter data are also reported because winter chilling is important for olive inflorescences formation, staminate flowers production, and bud break [28]. Minimum and maximum temperatures during bloom were in 2008 up to 1.5°C lower and in 2009 up to 1.3°C higher than the relative medium term averages. In both years, temperatures from January and February were lower compared to the means of the medium-term period differing less than 1.8°C in 2008 and up to 3.1°C in 2009. In spring, only the data recorded in April 2009 differ from the medium term averages and were 2°C higher.

**Figure 2:**
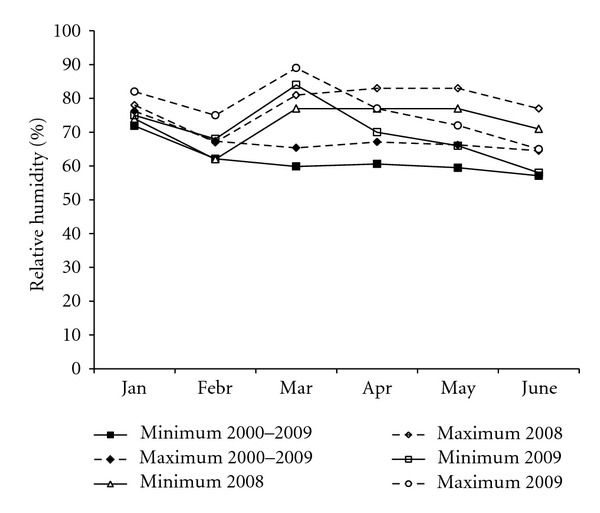
Comparison of Puegnago monthly means of maximum and minimum relative humidity from January to June in 2008 and 2009 compared to the long-term averages.

The monthly means of daily minimum and maximum relative humidity from January to June in 2008 and 2009 are shown in Figure 2 together with the medium term averages. Relative humidity in winter and spring of both years was higher compared to the medium-term averages. At bloom 2008, the monthly data were approximately 15% higher than the medium term averages, whereas in May 2009 the means were 6% higher and in June matched those of the medium-term averages.

### 3.2. Self-Compatibility and Cross-Compatibility

**Figure 3:**
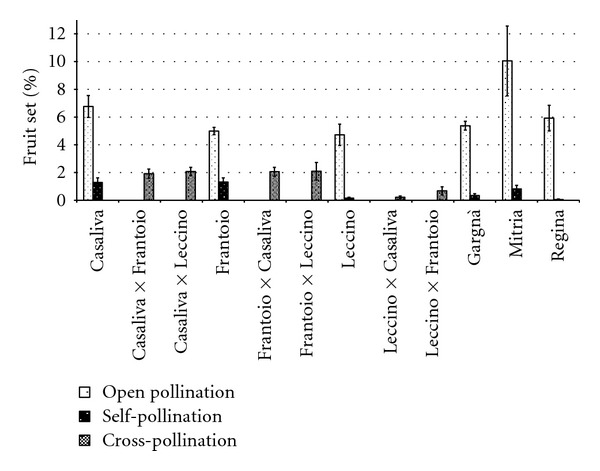
Effect of open, cross- and self-pollination on fruit set of six olive cultivars. Values are means with standard errors (*n* = 10).

In all studied cultivars, fruit set following self-pollination was significantly lower than from open pollination (Figure 3). Self-pollinated “Frantoio” and “Casaliva” showed the highest values (1.3%), while “Regina” had almost no fruits and in “Leccino” the production was negligible. Fruit set after open pollination was similar in all the cv tested, except in “Mitria,” showing a significantly higher level. In “Casaliva,” “Frantoio,” and “Leccino,” it gave percentages of 7%, 5%, and 5%, respectively.

The ISI ranged from 0.264 in “Frantoio” and 0.191 in “Casaliva,” the more self-compatible cultivars, to 0.034 and 0.008 in “Leccino” and “Regina,” respectively. Therefore, all cultivars showed low levels of self-fertility.

**Figure 4:**
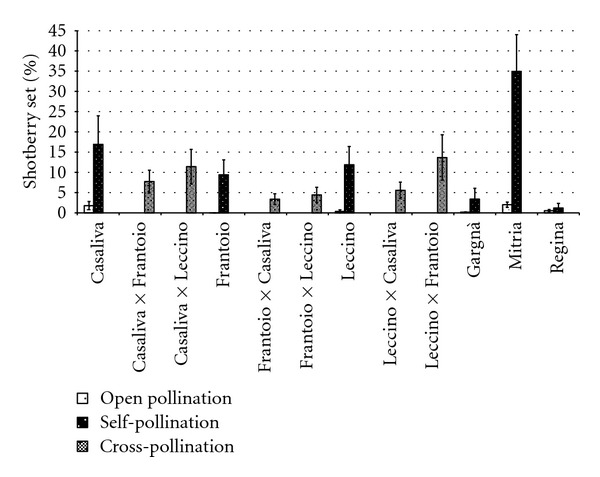
Effect of open, cross- and self-pollination on parthenocarpic fruit set of six olive cultivars. Values are means with standard errors (*n* = 10).

Except for “Regina,” shotberry production was significantly lower after open than after self-pollination (Figure 4). In open pollinated “Frantoio” and “Gargnà,” shotberry set was below 0.07%, whereas in “Mitria” it reached the highest value (2%) with a significantly higher amount also after selfing (34.95%).

Reciprocal crosses of “Casaliva,” “Frantoio,” and “Leccino” produced no significant increases over self-pollination in fruit set (Figure 3). Fruit set of crossed “Casaliva” and “Frantoio” was about 2%, and set percentages of selfed “Casaliva” and “Frantoio” were 35% lower. In “Leccino,” set after self-pollination was very low (0.16%) and after cross-pollination was below 1%. Consequently, ISI values were generally high ranging from 0.74 for “Leccino” relative to “Casaliva” pollen to 0.62 for the reciprocal cross. Only for “Leccino,” following cross-pollination with “Frantoio” pollen, ISI was 0.23, that is, “Frantoio” pollen induced more than a 4-fold increase in the fruit set of “Leccino” (0.69%) as compared to selfing. Nevertheless, also this pollination treatment did not differ significantly from self-pollination. Open pollination produced always higher rates of fruit set over cross-pollination (Figure 3).

Shotberries after cross-pollination were abundant, and no significant differences were detected with respect to self-pollination. “Casaliva,” “Frantoio,” and “Leccino” produced less shotberries after open pollination than after all cross combinations (Figure 4), but due to a high degree of variability in shotberry set recorded for all cultivars, only “Leccino” with “Frantoio” pollen had significantly higher amount of shotberries compared to open pollination (percentage set 13.7% and 0.4%, resp.).

### 3.3. Effect of B Foliar Application on Fruit Set

**Figure 5:**
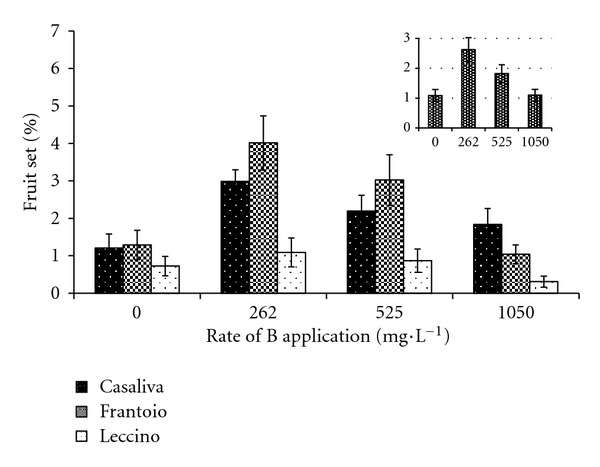
Effect of B foliar applications on fruit set in “Casaliva,” “Frantoio,” and “Leccino.” Internal box: average effect. Values are means with standard errors (*n* = 10).

Response to B treatment was different among cultivars. In “Frantoio,” at 262 and 525 mg·L^−1^ B increased significantly fruit set by 211% and 134%, respectively, whereas in “Casaliva” this effect was found only at 262 mg·L^−1^, with an increase of 147% (Figure 5). No significant differences in fruit set were detected in “Leccino” after any treatment. Irrespective of cultivar, the highest rate of B (1050 mg·L^−1^) showed the lowest fruit set and did not differ from the control. Furthermore, the highest set occurred at 262 mg·L^−1^. “Controlled” open pollination in control samples yielded 1.2%, 1.3%, and 0.8% fruit set for “Casaliva,” “Frantoio,” and “Leccino,” respectively.

**Figure 6:**
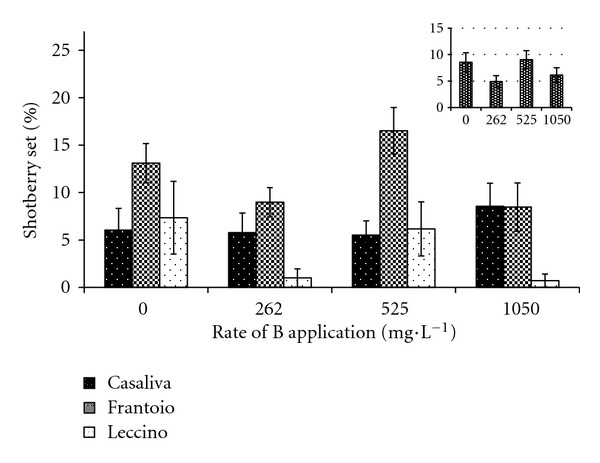
Effect of B foliar applications on parthenocarpic fruit set in “Casaliva,” “Frantoio,” and “Leccino.” Internal box: average effect. Values are means with standard errors (*n* = 10).

B foliar application did not significantly affect shotberry set. Furthermore, no trend toward increased or decreased shotberry set was detectable across different B treatments (Figure 6). Set ranged from 5.5% to 8.6% for “Casaliva” and reached 7.3% and 16.5% for “Leccino” and “Frantoio,” respectively.

### 3.4. Effect of B Foliar Application on *In Vitro* Pollen Germination

**Figure 7:**
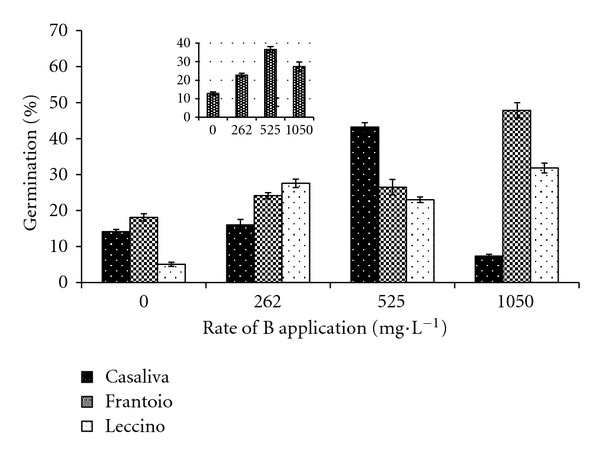
Effect of B foliar applications on *in vitro* pollen germination in “Casaliva,” “Frantoio,” and “Leccino.” Internal box: average effect. Values are means with standard errors (*n* = 10).

Foliar applied B resulted in significantly higher *in vitro* pollen germination rates. Cultivars showed different thresholds; in “Leccino” and “Frantoio” a significant effect was already detected at 262 mg·L^−1^, whereas in “Casaliva” only at 525 mg·L^−1^ (Figure 7). In “Leccino” and “Frantoio,” the highest germination rates occurred at maximum B concentration: 32% and 48%, that is, 6.3 and 2.6 times higher than the control, respectively. However, in “Casaliva” B at 525 mg·L^−1^ caused the highest percentage of germination, 3-fold higher with respect to the control, whereas maximum B level inhibited the process.

## 4. Discussion

Fruit set following self-pollination was consistently lower than that obtained from cross- and open pollination, as reported in many other works in the Mediterranean region [14, 29, 30]. Only in selfed “Casaliva” and “Frantoio” fruit set exceeded 1% and could result in adequate commercial crops. Open pollination increased olive setting by 275% in Frantoio, the cultivar with the lowest incompatibility index. According to Moutier et al. [30], “Frantoio” and “Casaliva” are classified as partially self-compatible (15–30% fruit set for selfed trees relative to open pollinated trees) and all other cultivars as highly self-incompatible (fruit set below 15% compared to open pollination). These results confirm, therefore, the presence of self-incompatibility in most Italian olives cultivars, as reported by other authors [8]. There are conflicting reports about self-incompatibility in “Frantoio,” ranging from highly self-incompatible [31] to self-compatible [32]. The discrepancy in incompatibility response may be related to the different climatic conditions of the region where the other studies were performed. Moreover, several clones of “Leccino,” one of the most widespread cultivar-population of olive in central Italy, have been reported to be self-compatible [10]. On the contrary, our results in northern Italy show that fruit set in selfed “Leccino” is very low (about 0.15%) and did not change in different years (data not shown), thus showing a marked self-incompatibility. The occurrence of parthenocarpic fruitlets, known as shotberries, was significantly higher after self- than following open pollination. These results suggest that the lower the fertilization, the higher the shotberry production, in agreement with the findings of other authors [33–35]. Nevertheless, there is variability in shotberry set among different twigs of the same cultivar and no clear correlation between degree of self-incompatibility and shotberry production was found. In fact, the proportion of shotberries in self-pollination treatment could be affected not only by low fertilization but also by the tree vigour of each cultivar. Competition among fruits or between fruits and vegetative growth regions for assimilates and growth regulators may, therefore, have been responsible for different fruiting behaviour of the assessed cultivar. Abscission due to intense competition during early fruit growth proceeds until about 35–45 days after full bloom [36]. In this study, fruit set percentages have been recorded 90 days after bloom, when abscission of young fruitlets has ended and subsequent fruit drop is negligible [3]. The fruit set integrates the response both to pollination treatments and to competition among fruits, thus being influenced by source-sink relationships in the tree. In vigorous trees, when maximum vegetative growth is not concurrent with fruit growth, the supply of assimilates to the fruits is higher and less competition among fruits occurs. Therefore, more shotberries could persist on the trees though parthenocarpic fruits showed attenuated competition and less sink strength, in contrast to normal fruit [36].

Cross-pollination generally enhances fruit set of olives [37, 38]. A positive but not significant effect of controlled cross-pollination over self-pollination on fruit set was detectable in all the cultivars investigated in this study. The increase in fruit set after crossing with respect to selfing ranged from 35% to 325% for “Leccino” pollinated with “Casaliva” and “Frantoio” pollens, respectively. “Frantoio” showed cross-compatibility with both “Casaliva” and “Leccino” pollens. After Moutier [39], the two cultivars were acceptable pollinizers of “Frantoio,” as the fruit set was 41% of open pollination. These results are convergent with those obtained by Wu et al. [31], who showed a good combining ability of “Frantoio.” On the contrary, “Casaliva” and “Leccino” as pollen recipients showed lower cross-compatibility with “Frantoio” pollen, thus indicating that no reciprocity occurred.

The lesser effect on fruit set of controlled cross-pollination compared to open pollination could partly explain the differences between fruit set in the pollination experiments and the results obtained from control samples after B treatments. In boron experiments, open pollination was replaced by a “controlled” open pollination using a mixture of “Casaliva,” “Frantoio,” and “Leccino” pollen. Fruit set of control samples in these experiments gave percentages close to the averaged results obtained from controlled cross-pollination and self-pollination. Nevertheless, since the pollination and boron experiments were performed in subsequent years, a year-to-year deviation cannot be excluded. 

Foliar applications of B before anthesis consistently improved olive fruit set, with a 2.5- and 4-fold increase in “Casaliva” and “Frantoio,” respectively. This is in line with the much higher B requirement observed at the reproductive stage compared to normal vegetative growth [40] and with the high B concentrations found in reproductive organs, especially styles, stigma, and ovaries [41, 42]. The beneficial effects of foliar B treatments on fruit set have been reported for other trees species, including almond [18, 21], hazelnut [43], pecan [44], walnut [45], and pear [46]. Our results agree with the study of Perica et al. [47], who reported a significant positive effect of foliar applied B on olive fruit set. Fruit set increase after B sprays of open pollinated olive trees might be mainly related to a positive function of B on female reproductive organs or on fertilised developing fruits. The response to B treatments varied among olive cultivars, and fruit set increase was the highest in “Frantoio” but not significant in “Leccino.” Variability in cultivar responsiveness to B supply was also found in almond by Nyomora et al. [21], who attributed the enhanced response to B treatments to a higher cultivar sensitivity to changes in B tissue levels, rather than to higher B requirement.

The highest B concentration (1050 mg·L^−1^) was less effective in increasing fruit set, suggesting an optimal B concentration at which fruit set is maximized. The highest concentration may have been toxic for female reproductive organs, leading to inhibition of some physiological step involved in fertilisation and/or subsequent fruit development. B toxicity may involve complexing of ribonucleotides which causes metabolic disturbances, excess of cell wall cross-links and inhibition of cell wall expansion [48]. There may be also impairment of cell membrane functions, with disorders in transmembrane electrochemical potential and in transport of ions and metabolites [49], resulting in perturbation of the fertilisation process and developmental constraints. As in our trial, for many different fruits and nut crops the B concentrations that positively affect fruit set and yield range between 200 and 500 mg·L^−1^ [20, 21].

B is known to be required for pollination and fertilisation, and a role has been suggested also in the successive fruitlet retention. Competition for assimilates among developing fruits is suggested to be the main cause of abscission [3], whereas lack of fertilisation, although involved, is generally not the major cause of abscission [6]. The onset of ovary growth is the trigger for a selective fruitlet abscission, which affects mainly those pistils unable to reach a specific size [3]. B could also affect the sink strength of developing fruitlets as it has been involved in carbohydrate metabolism, sugar transport, and auxin turnover [49–51]. Furthermore, B deficiency causes an alteration in the expression of a wide range of genes involved in several physiological processes, including B uptake and translocation, maintenance of cell wall and membrane function, nitrogen assimilation, and plant stress response [52]. Interestingly, B sprays had no effect on shotberry set, suggesting that these parthenocarpic fruits did not strongly compete for resources allocation and did not take advantage of increased B tissue levels [39].

Moreover, B could play a part in female reproductive organs and in their interaction with pollen tube during his growth through the style and into the ovary. The physiological role of B, as borate, relies on the function as a cross-linking molecule involving reversible covalent ester bonds with cis-diols on either part of borate. Owing to the borate crosslinking of two monomers of rhamnogalacturonan II (RG II) in the pectin fraction of plant cell walls [53, 54], B plays a key role in assembly and mechanical properties of cell wall matrix [41, 55]. B is essential for pollen tube growth, and it is known to be incorporated into the pectins of pollen cell walls that contain RG II [56, 57]. As a consequence, foliar B applications can indirectly affect pollen tube growth increasing the B content in female reproductive structures. The adhesive pectic matrix in lily contains RG II, and pollen tube walls may bind the stylar matrix by means of these RG II borate crosslinks [58]. These events may be important in the fast guidance of pollen tubes through the style to the ovules.

B deficiency also causes defects in assembly and mechanical properties of cell walls and in structural and functional integrity of the plasma membrane [59].

In our experiments, foliar B application on field grown olive trees consistently enhanced *in vitro* pollen germination of all three tested cultivars, although the response varied among genotypes. Our results showed that the stimulation effect was different among cultivars. In “Frantoio,” there was a proportional increase of pollen germination at increasing B concentrations. In “Casaliva,” the maximum level of B resulted in an inhibition of pollen germination. In “Leccino,” there was a strong response to all the B treatments. 

These findings are in contrast with the results of Perica et al. [47], who found no *in vitro* pollen germination increase in olive trees treated with 246 to 737 mg·L^−1^ of B. Differences in initial endogenous B concentrations, different cv sensitivity to changes in B levels, or other environmental conditions may account for this discrepancy.

The effect of B on *in vitro* pollen germination did not exactly parallel its effect on fruit set. In “Leccino,” foliar B treatments did not influence significantly fruit set, whereas germination of pollen of B sprayed trees increased consistently at any B application rate, suggesting that pollen germination was not the limiting factor in “Leccino” fruit set. Moreover, the optimal level of B treatment is higher for pollen germination than for fruit set, and the greatest effect on “Casaliva” fruit set was observed at 262 mg·L^−1^ B, a concentration which did not influence pollen germination. These results suggest that B levels in other tissues, probably in pistils, could affect olive sexual reproduction.

The implications of B in cell metabolism could account for its stimulatory effect on pollen germination and subsequent pollen tube growth. In our trial, both effects have been verified and beside the increase of germination, foliar B application did also greatly enhance pollen tube growth in all cultivars (data not shown). High B concentrations inhibiting pollen germination resulted also in a slower or inhibited pollen tube growth (data not shown).

In summary, our results highlight the importance of cross-pollination of olive cultivars for obtaining satisfactory fruit set in the environmental conditions of northern Italy and the beneficial effect of foliar B applications on male and female reproductive organs, possibly by affecting positively the fertilisation process and subsequent plant source-sink relations linked to fruitlet retention.
